# Modified peroral endoscopic septotomy using a thin-needle high-pressure waterjet knife for short-septum Zenker's diverticulum

**DOI:** 10.1055/a-2957-2389

**Published:** 2026-09-28

**Authors:** Ludovico Alfarone, Miriana Mercurio, Davide Massimi, Roberto De Sire, Roberta Maselli, Cesare Hassan, Alessandro Repici

**Affiliations:** 1 Department of Gastroenterology 9268 IRCCS Humanitas Research HospitalRozzanoItaly; 2 Department of Biomedical Sciences 437807 Humanitas UniversityMilanLombardyItaly

Flexible endoscopic septotomy is effective for Zenker’s diverticulum (ZD), but recurrence may occur following incomplete cricopharyngeal myotomy. 1​ Third-space techniques, including Zenker’s peroral endoscopic myotomy and peroral endoscopic septotomy (POES), were developed to achieve a more complete myotomy. 2​ POES provides direct septal access without proximal tunneling, achieving 100% technical and 95% clinical success in its initial series. 3​

An 80-year-old woman presented with dysphagia. Barium esophagogram showed a ZD of approximately 15 mm, confirmed at upper gastrointestinal endoscopy. The patient underwent POES under general anesthesia.

To avoid further narrowing of the limited working space by an initial submucosal cushion, we modified the conventional sequence of POES by performing a direct mucosal incision before submucosal injection ( Fig. 1 ). Mucosal incision, submucosal dissection and cricopharyngeal myotomy were performed with a novel thin-needle knife with high-pressure waterjet injection capability without device exchange (HYBRIDknife flex I-Type, 1.5 mm; ERBE Elektromedizin GmbH; Video 1 ). Its 1.5 mm needle electrode enabled precise cutting, while the integrated high-pressure waterjet injection system ensured on-demand submucosal injection at each step. 4​ In short-septum diverticula, myotomy should be stopped once the cricopharyngeal muscle has been completely divided at the base of the septum, with no residual muscular bridge. Intraprocedural bleeding was managed with saline-immersion knife-tip coagulation without using hemostatic forceps. 5​ Complete closure of mucosal incision was performed using standard clips. No adverse events were observed.

**Fig. 1:**
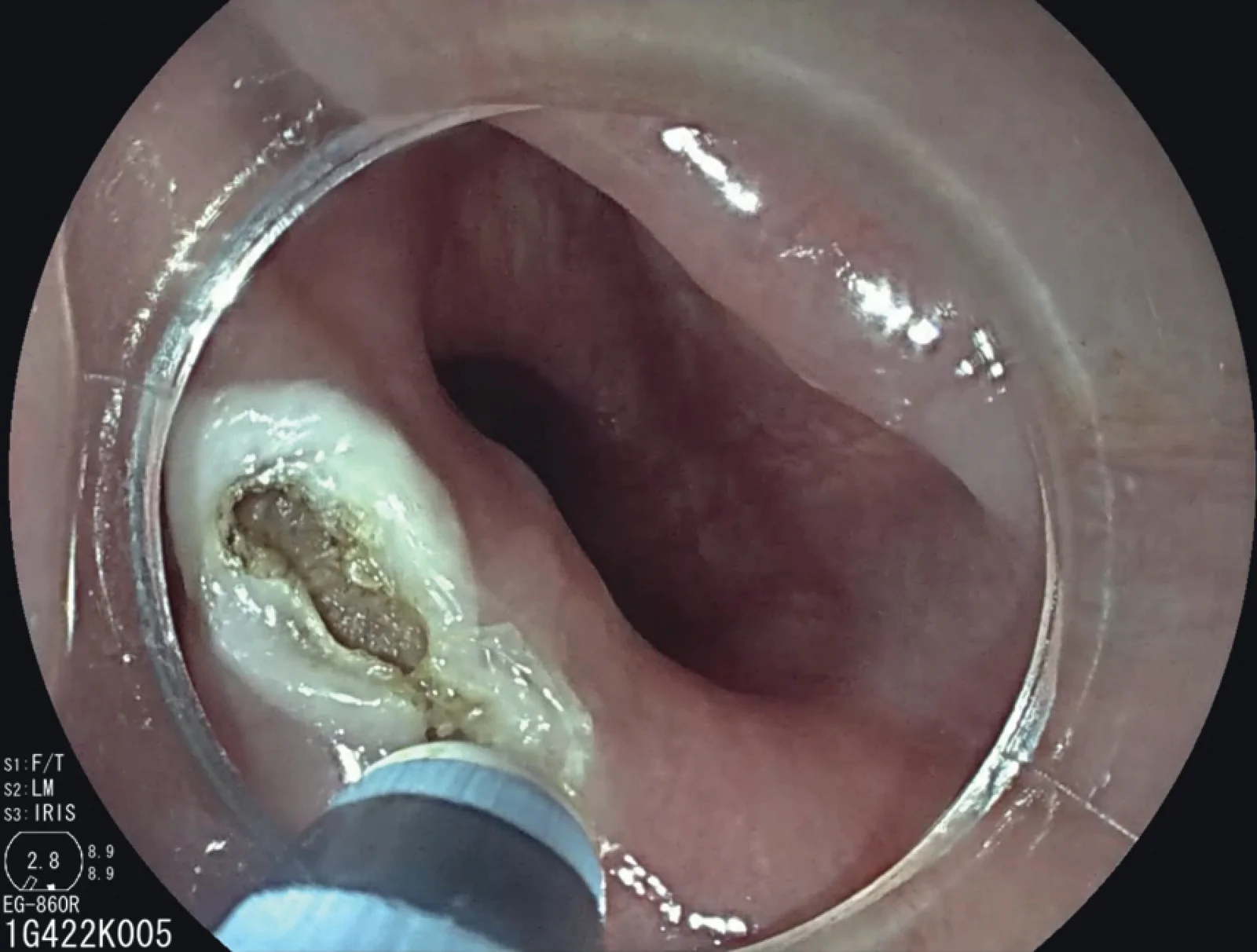
An endoscopic view of the mucosal incision of the septum performed before submucosal injection.

**Video 1** Modified peroral endoscopic septotomy using an incision-first approach and a thin-needle high-pressure waterjet knife for short-septum Zenker’s diverticulum.


Complete cricopharyngeal myotomy was achieved, with dysphagia resolution at 6 months follow-up.

To our knowledge, this is the first reported POES performed with this device. This case demonstrates that this novel thin-needle knife combining cutting precision and waterjet injection system in a single device enabled a seamless modified POES workflow and could be a viable option for short-septum ZD.

## Declaration of GenAI Use

The authors confirm that no generative artificial intelligence was used in the preparation of this manuscript.

Endoscopy_UCTN_Code_TTT_1AO_2AP
